# Food Allergy Knowledge and Attitudes among School Nurses in an Urban Public School District

**DOI:** 10.3390/children2030330

**Published:** 2015-07-21

**Authors:** Sarah Twichell, Kathleen Wang, Humaira Robinson, Maria Acebal, Hemant Sharma

**Affiliations:** 1 Division of Endocrinology, Boston Children’s Hospital, 300 Longwood Avenue, Boston, MA 02115, USA; E-Mail: Sarah.Twichell@childrens.harvard.edu; 2 Division of Allergy and Immunology, Children’s National Health System, 111 Michigan Avenue NW, Washington, DC 20010, USA; E-Mails: KaWang@childrensnational.org (K.W.); HuRobins@childrensnational.org (H.R.); 3 Safe@Schools Partners; Food Allergy Research & Education (FARE), 7925 Jones Branch Drive #1100, Tysons, VA 22102, USA; E-Mail: mlacebal@gmail.com

**Keywords:** food allergy, anaphylaxis, school nurse, knowledge, attitudes

## Abstract

Since food allergy knowledge and perceptions may influence prevention and management of school-based reactions, we evaluated them among nurses in an urban school district. All District of Columbia public school nurses were asked to anonymously complete a food allergy knowledge and attitude questionnaire. Knowledge scores were calculated as percentage of correct responses. Attitude responses were tabulated across five-point Likert scales, ranging from strongly disagree to strongly agree. The knowledge questionnaire was completed by 87% of eligible nurses and the attitude questionnaire by 83%. The mean total knowledge score was 76 ± 13 with domain score highest for symptom recognition and lowest for treatment. Regarding attitudes, most (94%) felt food allergy is a serious health problem, for which schools should have guidelines (94%). Fewer believed that nut-free schools (82%) and allergen-free tables (44%) should be implemented. Negative perceptions of parents were identified as: parents of food-allergic children are overprotective (55%) and make unreasonable requests of schools (15%). Food allergy knowledge deficits and mixed attitudes exist among this sample of urban school nurses, particularly related to management of reactions and perceptions of parents. Food allergy education of school nurses should be targeted to improve their knowledge and attitudes.

## 1. Introduction

Food allergy is a significant health concern among children in the United States. Recent studies of self-reported food allergy show prevalence ranging from 3.9% to 8.0% [1,2,3]. The largest study of food allergy prevalence in children found that 8%, or approximately 1 in 13 children in the United States, have a food allergy. Furthermore, there is evidence that the prevalence of food allergy has been increasing over the past 20 years [4,5,6].

The increasing prevalence of pediatric food allergy has been observed specifically in schools as well. Among the 400 American elementary school nurses surveyed, nearly half reported an increase in students with food allergies over a five-year period, and more than one-third reported having 10 or more students with food allergies [7]. They considered the challenge of managing food allergies equivalent to other serious health conditions, such as diabetes.

Several studies have evaluated the characteristics of food allergic reactions in schools [8,9,10]. Not surprisingly, a number of food allergic reactions and up to two-thirds of food allergy-related deaths among children may occur in the school setting [11,12]. Much of the prior work surrounding food allergy and schools has focused on the implementation of comprehensive food allergy and anaphylaxis management policies. Also, tools have been developed to aid school nurses in educating school staff regarding food allergy [13,14]. Recent studies have investigated food allergy knowledge among school administrators and teachers [15,16,17], but little emphasis has been placed on knowledge among school nurses, who directly provide health care services. A recent needs assessment among school nurses indicated that though most nurses identified their baseline knowledge of food allergies as strong or very strong, they were highly interested in increasing their knowledge. Specifically, they had interest in learning how to teach school staff about food allergies and develop food allergy management plans [18,19].

Knowledge and attitudes may influence prevention and management of school-based food allergy reactions. Therefore, the goal of this study was to assess knowledge and attitudes towards food allergy among school nurses in an urban public school setting. The study found that knowledge deficits and mixed attitudes exist among these school nurses.

## 2. Results

The knowledge questionnaire was completed by 171 of 196 eligible nurses (87%), and the attitude questionnaire by 162 of 196 (83%). All of the nurses in the study population were female. The majority of nurses reported knowing someone with a food allergy, and approximately one-quarter reported having a food allergy themselves, which agrees with prior findings of an overestimation of self-reported food allergy in the general population [20]. More than half reported having treated a student for a food allergic reaction in the past year, while less than half received prior education about food allergies (Table 1).

**Table 1:** Demographic characteristics of school nurses.

Characteristic	Sample Population (N = 162), n (%)
Female Gender	162 (100)
Nurse has food allergy	33 (24)
Nurse knows someone with food allergy	120 (87)
Nurse previously received information about food allergy	58 (42)
Nurse treated student for food-allergic reaction within past year	87 (59)

### 2.1. Knowledge

**Figure 1:**
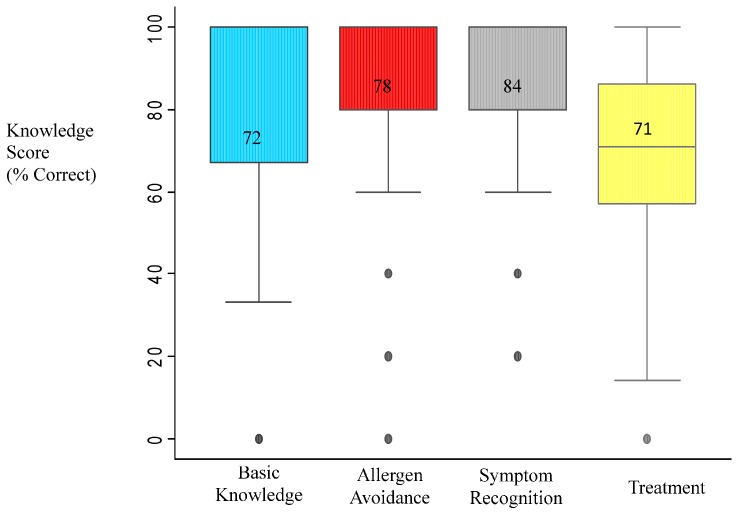
Knowledge scores by content domain.

The mean total knowledge score was 76.7 (SD = 13.5). The lowest scores were for questions related to treatment and basic knowledge, while the highest scores were for questions addressing symptom recognition (Figure 1). There were no significant relationships between any of the demographic characteristics and knowledge scores. Knowledge strengths and weaknesses were identified (Table 2 and Table 3, respectively). Most of the deficits were related to items assessing knowledge of appropriate treatment of reactions. For example, only half of nurses correctly responded that epinephrine is not an extremely dangerous medication, less than half knew that a second dose of epinephrine could safely be administered for persistent symptoms, and less than two-thirds knew that antihistamines should not always be the first medication administered. Another knowledge gap was related to allergen avoidance, namely whether hand sanitizer adequately removes food allergens from hands (39% correct).

**Table 2:** Food allergy knowledge strengths.

Item	Prevalence of Correct Response
**Allergen Avoidance**	
A package with a precautionary label stating “processed on shared equipment with milk” but not listing milk as an ingredient is safe for a milk-allergic child. (False)	93%
A small bite of food is not enough to cause a life-threatening reaction. (False)	91%
If a salad is topped with walnuts, you can safely feed it to a walnut-allergic person if you remove the walnuts. (False)	96%
**Symptom Recognition**	
A person can die within minutes from a food allergic reaction. (True)	93%
Anaphylaxis can cause shock, a sudden and dangerous drop in blood pressure. (True)	95%
**Treatment**	
Epinephrine should be given to treat an allergic reaction only if a student has lost consciousness, but not before. (False)	92%
Parents whose peanut-allergic child attends a peanut-free school do not need to supply the school with epinephrine or an emergency action plan. (False)	94%

**Table 3:** Food allergy knowledge weaknesses.

Item	Prevalence of Correct Response
**Basic Knowledge**	
The course of a food allergic reaction is predictable if you know the allergic symptoms a student has experienced in the past. (False)	47%
**Allergen Avoidance**	
To reduce cross-contamination from food residue left on hands after eating, use of an anti-bacterial hand sanitizer is recommended. (False)	39%
**Treatment**	
You may safely administer a second dose of epinephrine if allergic symptoms have not improved after 10 min of receiving the first dose. (True)	39%
Epinephrine is an extremely dangerous drug with many potentially harmful side effects. (False)	49%
Benadryl or another antihistamine should always be the first medication given when a student is having a food-allergic reaction. (False)	63%

### 2.2. Attitudes

As would be expected, almost all school nurses agreed or strongly agreed that food allergy is a serious health problem, for which schools should have guidelines (Table 4). While most agreed that nut-free schools help to keep children with food allergies safe, less than half felt that students with food allergies should have allergen-free tables at their schools. Significantly more nurses agreed with allergen-free tables if they had received prior food allergy education. Agreement with nut-free schools and allergen-free tables were also significantly related to a belief that food-allergic students encounter difficulty eating at school and eating out (Table 5).

**Table 4:** School nurse attitudes towards food allergy.

Item	Prevalence Agree/Strongly Agree
**General Food Allergy Beliefs**	
I think food allergy is a serious problem for children in the United States.	94%
**School Policy Attitudes**	
Schools should have guidelines for managing food allergy reactions in students.	94%
Nut-free schools help to keep students with nut allergy safe, and should be implemented in my school.	82%
Students with food allergy should have special allergen-free tables available so they can safely eat at school.	44%
**Perceptions of Student Impact**	
It is hard for students with food allergy to safely eat out.	53%
It is hard for students with food allergy to safely eat at school.	43%
Students with food allergy tend to worry a lot about their condition.	40%
Students are teased/bullied about their food allergy in the school setting.	32%
**Perceptions of Parents**	
Parents of food-allergic children tend to be more overprotective than parents of children with other chronic illnesses.	55%
Parents of food-allergic children make unreasonable requests of school personnel.	15%

**Table 5:** Predictors of nurse school policy attitudes.

Subgroup Characteristic	Agreement with Nut-free Schools *n* (%)	*p* Value	Agreement with Allergen-Free Tables *n* (%)	*p* Value
Overall population	130 (82)		68 (44)	
Nurse previously received information about food allergy.				
Yes	44 (77)		32 (56)	
No	64 (84)	0.30	25 (33)	0.01 *
Nurse believes it is hard for students with food allergy to eat out.				
Yes	72 (89)		41 (53)	
No	54 (74)	0.02 *	26 (36)	0.04 *
Nurse believes it is hard for students with food allergy to eat at school.				
Yes	60 (94)		35 (56)	
No	63 (72)	<0.01 *	31 (36)	0.02 *
				

(*) statistically significant

Regarding the impact of food allergy on a student’s quality of life, slightly less than half felt that students with food allergy worry about their condition and only one-third believed students were teased or bullied about their food allergy. Concerning perceptions of parents, more than half of nurses agreed parents of food-allergic children are more overprotective than parents of children with other chronic illnesses. About one in seven nurses felt parents of food-allergic students make unreasonable requests of school personnel (Table 4). Nurses’ perceptions of parents and perceptions of students were significantly associated with each other. Nurses who felt that parents of food-allergic children were more overprotective were significantly more likely to believe that students worried about their food allergy and were teased or bullied. The same associations were observed among nurses who felt that parents make unreasonable requests of schools (Table 6).

**Figure 2:**
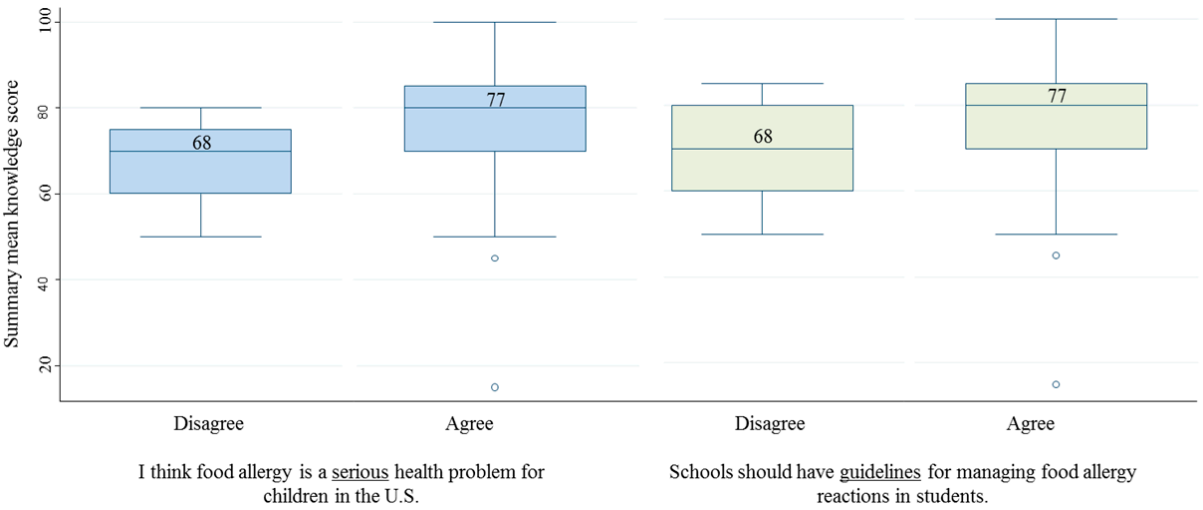
Knowledge scores by nurse attitudes.

Mean summary knowledge scores were compared for each attitude statement between those nurses who agreed *versus* disagreed. Scores were significantly higher among nurses who felt that food allergy was a serious problem and also among those who thought schools should have food allergy guidelines (Figure 2). None of the other attitude statements were significantly associated with knowledge scores.

**Table 6:** Relationships of nurses’ parent and student perceptions.

Subgroup Characteristic	Agreement that Food-Allergic Students Worry *n* (%)	*p* Value	Agreement that Food-Allergic Students Are Teased/Bullied *n* (%)	*p* Value
Overall population	62 (40)		51 (32)	
Nurse believes parents of food-allergic children are more over-protective than others.				
Yes	41 (48)		36 (41)	
No	20 (29)	0.02 *	15 (22)	0.01 *
Nurse believes parents of food-allergic children make unreasonable requests of school.				
Yes	17 (74)		12 (55)	
No	44 (34)	<0.01 *	38 (29)	0.02 *

(*) statistically significant

## 3. Discussion

This study evaluated the knowledge and attitudes of District of Columbia public school nurses regarding food allergies. These baseline data provide insights into knowledge gaps and attitudes towards students with food allergies, allowing for the development of targeted educational programs designed to address these knowledge gaps and attitudes.

### 3.1. Knowledge

Overall, the greatest knowledge deficits were related to general food allergy knowledge and treatment of food allergies, which is concerning since morbidity and mortality related to food allergy is consistently linked with delayed treatment with epinephrine. In two separate reviews of anaphylaxis-related deaths, only 6–13% of individuals received epinephrine in a timely fashion [21,22]. Deaths from food allergy have occurred in the school setting, again often related to delayed epinephrine administration [12]. It is therefore imperative that students with food allergies have reliable access to epinephrine at school and that school nurses and other school healthcare providers are knowledgeable about recognizing and treating food allergic reactions. In this study, one of the knowledge deficits is not recognizing epinephrine as a safe medication for treatment of anaphylaxis, but rather believing that epinephrine is a very dangerous medication with harmful side effects. Many nurses also answered that an antihistamine, rather than epinephrine, should always be the first-line medication for treating food-allergic reactions and many did not know that it is safe to give a second dose of epinephrine if symptoms persist. These findings are concerning given that such knowledge gaps may lead to delayed or inappropriate treatment of food-induced anaphylaxis. Since food allergies are prevalent and reactions are likely to happen in the school setting [2], it is critical that school nurses and health providers are aware of appropriate treatment for children with food allergies. Given that areas with the greatest gap in knowledge related to treatment, this topic would be a key area to highlight during food allergy education for school nurses.

Another area of knowledge deficit related to whether hand sanitizer is adequate to eliminate food allergens from hands, which over half of nurses incorrectly believed to be true. Knowledge about hand hygiene is essential not just for school nurses, but also for staff who may be handling food allergens. Given the role of school nurses as health educators of other school personnel, ensuring that nurses are knowledgeable about how to appropriately eliminate allergens from surfaces may help decrease the frequency of food allergic reactions.

### 3.2. Attitudes

This study also provides one of the first published reports of school nurse attitudes towards food allergies. Overall, nurses were well aware of both the seriousness of food allergies and the need for schools to have clear guidelines relating to treatment of food allergies, but less than half felt that students with food allergies should have allergen-free tables at their schools. In addition, despite this acknowledgement of the potential significance of food allergies, almost half of nurses did not think that it was difficult for children with food allergies to either eat out or eat in school. The disparity between beliefs that policies should be in place and actual support for these policies has been observed in other studies. For example, in a study of attitudes among the general public, most believe that schools should have a policy to keep food-allergic children safe, yet also oppose specific school policies such as banning peanuts and tree nuts [23]. It is not entirely clear why there is a gap between perceptions of the severity of food allergy and a direct acknowledgement of the challenges of living with food allergies. This may relate to an incomplete understanding of a potentially life-threatening food allergy, as in this study a larger than expected proportion of nurses reported that they themselves had a food allergy and overall baseline knowledge about food allergy was one of the lower scoring knowledge sections. It should be noted, however, that policies banning certain foods, such as peanuts, from schools have been shown to not be fully effective as reactions have been reported in schools with active bans [9]. Therefore, the 18% of nurses in this study who disagreed with nut-free schools as an effective policy for preventing reactions are actually correct given this evidence and their opposition to nut-free policies could certainly be justified.

Nurses’ attitudes about bullying and teasing associated with food allergies was also assessed. Several recent studies found that a significant number of children suffering from food allergies encounter bullying, teasing, or harassment at school, ranging from one fourth to one third of students [24,25]. Notably, in one study, 21% of those bullied or teased named the perpetrators as teachers or school staff [25]. Bullying has been shown to be associated with lower quality of life and distress in these children [24]. Despite these important implications, less than one third of nurses in this study believed that students were teased or bullied about their food allergies at school. Increasing awareness among school nurses of the prevalence of bullying and teasing of children with food allergies in the school setting may help minimize these occurrences and help improve quality of life for these children. 

There was also a belief that parents of children with food allergies were more overprotective than parents of children with other chronic illnesses, with over half of nurses agreeing with this statement. Although fewer nurses (15%) felt that parents of food-allergic children make unreasonable requests of schools, this finding is also somewhat concerning. These attitudes towards parents of children with food allergy provide an interesting perspective on the challenges facing both nurses and families in the school setting.

Despite these beliefs, it is encouraging that prior food allergy education was a significant predictor of nurses’ support of school accommodations for food allergy, namely allergen-free tables. This suggests that the attitudes of nurses toward food allergy may be modified by education. Also, it appeared that the nurses’ insight into the quality-of-life challenges faced by students with food allergy when eating out was an additional predictor of agreement with allergen-free tables. Therefore, food allergy education of school nurses should include discussion of the quality-of-life implications of the disease, which may help improve attitudes towards students and parents of students with food allergies.

### 3.3. Limitations

This study evaluated school nurse knowledge and attitudes towards food allergies; while the data provide one of the first large samples of school nurse perceptions and attitudes about food allergy, there are some key limitations to this study. We did not collect extensive demographic data on the nurses; it would have been useful to have more data regarding number of years in practice and duration of time in the public school system. These data were also collected in a single urban school system with a fairly uniform set of regulations regarding medications and treatment plans and with school nurses present in every school. The findings are likely not generalizable to all school systems, particularly those without school nurses on site.

## 4. Materials and Methods

School nurses with either a Registered Nurse (RN) or Licensed Practical Nurse (LPN) license working in public or public charter schools in the District of Columbia (*n* = 196) were asked to anonymously complete food allergy knowledge and attitude questionnaires at a Summer Nurse Institute hosted by Children’s National Medical Center. The study was approved by the Children’s National Medical Center Institutional Review Board (IRB).

The questionnaire was adapted from a tool designed for the Safe@School™ CARE curriculum (http://store.foodallergy.org/ProductDetails.asp?ProductCode=SAS), provided by Food Allergy Research & Education (FARE). The questionnaire was developed by literature review and expert panel input, and was previously utilized in a study of school personnel and child care providers [26]. The 20-question true/false knowledge questionnaire evaluated food allergy knowledge across multiple domains including general knowledge about food allergy, avoidance of allergens, recognition of symptoms, and treatment of allergic reactions.

A 10-question food allergy attitude questionnaire was also administered. The items were developed based on literature review [27], expert panel input, and focus groups. Four content domains were assessed, including general food allergy beliefs, school policy, perceptions of students, and perceptions of parents. Response options were across a five-point Likert scale, ranging from “strongly disagree” to “strongly agree.”

A series of demographic questions also evaluated whether participants knew someone who had a food allergy and whether they had received prior food allergy training or recently treated a student with a food allergy.

Regarding statistical analysis, incomplete questionnaires were excluded from analysis. Summary and domain knowledge scores were calculated as a percentage of correct responses (range 0–100), and mean knowledge scores were calculated. The Wilcoxon rank sum test was used to measure associations between knowledge scores and demographic characteristics. Knowledge strengths and weakness were identified based on the prevalence of correct responses >90% and <65%, respectively. Attitude responses were tabulated across a five-point Likert scale to assess the proportion of participants who agreed or strongly agreed with attitude statements. Cross tabulation, chi square tests, and logistic regression were used to examine relationships between item responses and nurse characteristics and food allergy knowledge scores. Analysis was performed with StataSE 9.0.

## 5. Conclusions

This study represents one of the first published analyses of school nurse attitudes and knowledge regarding food allergy. The knowledge gaps identified by this study should allow for development of more structured school nurse education programs, with the ultimate goal of increasing the safety of children with food allergies in school. In addition, this study identified varied attitudes of school nurses towards children with food allergy, and ideally a training program would also seek to address quality-of-life issues for children with food allergy that may not be fully appreciated by school health providers.

## Supplementary Files

Supplementary File 1
