# Supplementary material for: Food Allergy Knowledge and Attitudes among School Nurses in an Urban Public School District
Source: Children (Basel). 2015 Jul 21;2(3):330–41. doi: 10.3390/children2030330 (PMC4928767; doi:10.3390/children2030330)

Table 1: Demographic characteristics of school nurses

Characteristic	Sample Population (N=162), n (%)
Female Gender	162 (100)
Nurse has food allergy	33 (24)
Nurse knows someone with food allergy	120 (87)
Nurse previously received information about food allergy	58 (42)
Nurse treated student for food-allergic reaction within past year	87 (59)

Figure 1: Knowledge scores by content domain

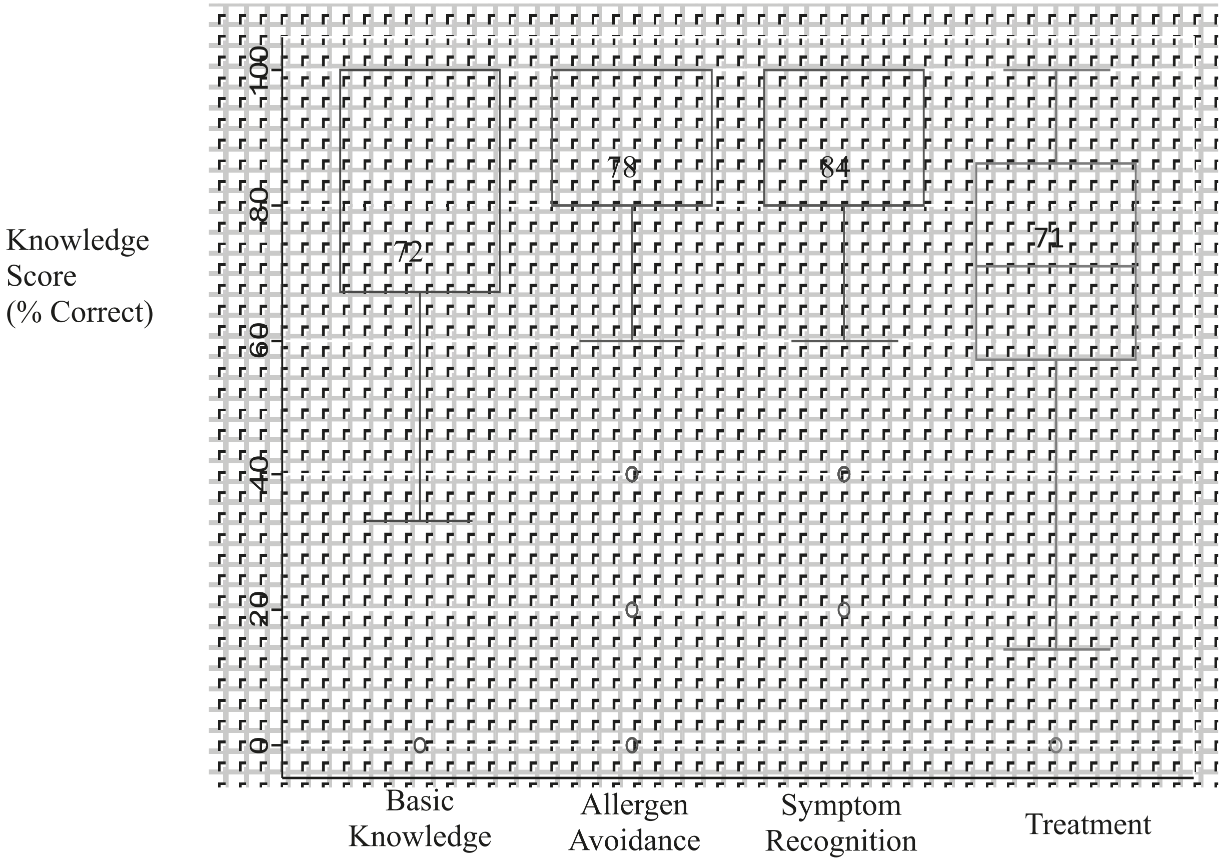

Table 2: Food Allergy Knowledge Strengths

Item	Prevalence of Correct Response
**Allergen Avoidance**	
A package with a precautionary label stating "processed on shared equipment with milk" but does not list milk as an ingredient is safe for a milk-allergic child. (False)	93 %
A small bite of food is not enough to cause a life-threatening reaction. (False)	91 %
If a salad is topped with walnuts, you can safely feed it to a walnut-allergic person if you remove the walnuts. (False)	96 %
**Symptom Recognition**	
A person can die within minutes from a food allergic reaction. (True)	93 %
Anaphylaxis can cause shock, a sudden and dangerous drop in blood pressure. (True)	95 %
**Treatment**	
Epinephrine should be given to treat an allergic reaction only if a student has lost consciousness, but not before. (False)	92 %
Parents whose peanut-allergic child attends a peanut-free school do not need to supply the school with epinephrine or an emergency action plan. (False)	94 %

Table 3: Food Allergy Knowledge Weaknesses

Item	Prevalence of Correct Response
**Basic Knowledge**	
The course of a food allergic reaction is predictable if you know the allergic symptoms a student has experienced in the past. (False)	47 %
**Allergen Avoidance**	
To reduce cross-contamination from food residue left on hands after eating, use of an anti-bacterial hand sanitizer is recommended. (False)	39 %
**Treatment**	
You may safely administer a second dose of epinephrine if allergic symptoms have not improved after 10 minutes of receiving the first dose. (True)	39 %
Epinephrine is an extremely dangerous drug with many potentially harmful side-effects. (False)	49 %
Benadryl or other antihistamine should always be the first medication given when a student is having a food-allergic reaction. (False)	63 %

Table 4: School nurse attitudes towards food allergy

Item	Prevalence Agree/Strongly Agree
**General Food Allergy Beliefs**	
I think food allergy is a serious problem for children in the United States.	94 %
**School Policy Attitudes**	
Schools should have guidelines for managing food allergy reactions in students.	94 %
Nut-free schools help to keep students with nut allergy safe, and should be implemented in my school.	82 %
Students with food allergy should have special allergen-free tables available so they can safely eat at school.	44 %
**Perceptions of Student Impact**	
It is hard for students with food allergy to safely eat out.	53 %
It is hard for students with food allergy to safely eat at school.	43 %
Students with food allergy tend to worry a lot about their condition.	40 %
Students are teased/bullied about their food allergy in the school setting.	32 %
**Perceptions of Parents**	
Parents of food-allergic children tend to be more overprotective than parents of children with other chronic illnesses.	55 %
Parents of food-allergic children make unreasonable requests of school personnel.	15 %

Table 5: Predictors of Nurse School Policy Attitudes

Subgroup Characteristic	Agreement with Nut-free Schools n (%)	p value	Agreement with Allergen-Free Tables n (%)	p value
Overall population	130 (82)		68 (44)	
Nurse previously received information about food allergy				
Yes	44 (77)	0.30	32 (56)	0.01 *
No	64 (84)		25 (33)	
Nurse believes it is hard for students with food allergy to eat out				
Yes	72 (89)	0.02 *	41 (53)	0.04 *
No	54 (74)		26 (36)	
Nurse believes it is hard for students with food allergy to eat at school				
Yes	60 (94)	<0.01 *	35 (56)	0.02 *
No	63 (72)		31 (36)	

Table 6: Relationships of Nurses' Parent and Student Perceptions

Subgroup Characteristic	Agreement that Food-Allergic Students Worry n (%)	p value	Agreement that Food-Allergic Students are Teased/Bullied n (%)	p value
Overall population	62 (40)		51 (32)	
Nurse believes parents of food-allergic children are more over-protective than others				
Yes	41 (48)	0.02 *	36 (41)	0.01 *
No	20 (29)		15 (22)	
Nurse believes parents of food-allergic children make unreasonable requests of school				
Yes	17 (74)		12 (55)	
No	44 (34)	< 0.01 *	38 (29)	0.02 *

Figure 2: Knowledge Scores by Nurse Attitudes

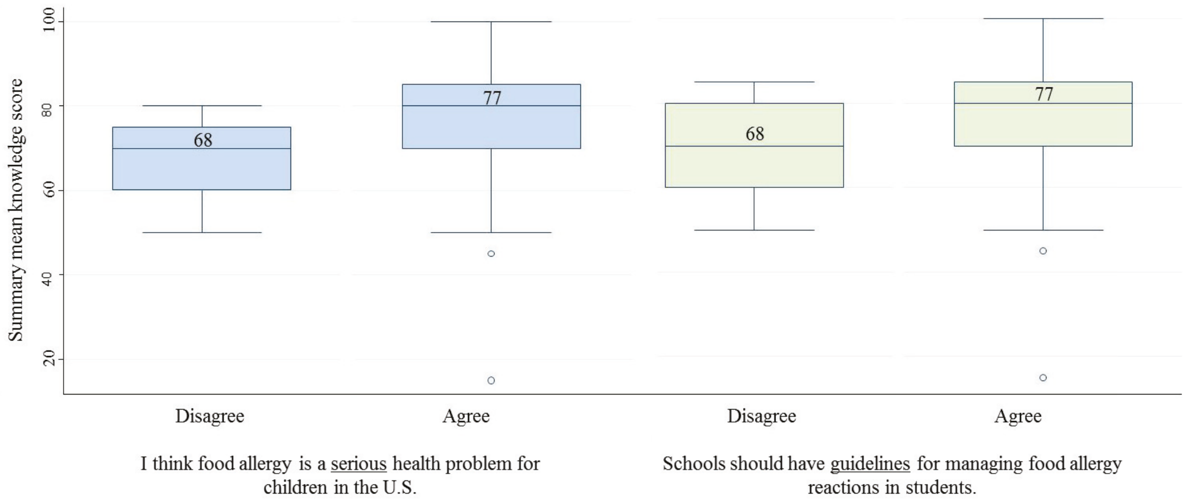

Supplement: Supplementary File 1 [file children-02-00330-s001.pdf]
